# A Detail of an Experiment, with Observations, on the Oleum Terebinthinœ, Regarding Its Effect on the Animal Economy

**Published:** 1821-07

**Authors:** James Copland


*A Detail of an Experiment, with Observations, on the Oleum Terebinthinæ, regarding its Effect on the Animal Economy.* By JAMES COPLAND, M.D.

I BELIEVE nothing can more effectually promote the advancement of our knowledge of the operations of the animal economy, and the mutual influence exerted between the several systems of the body, than faithfully detailed experiments, made not only upon the lower animals, but also upon man himself, when they can be resorted to with safety.

Were such inquiries more frequently instituted, I am confident we should be enabled to resort to remedial agents in disease with greater effect; having previously acquired more precise ideas respecting their mode of operation upon the various textures of the system. Hence, a double advantage would evidently be derived from a prosecution of such experimental inquiries: our knowledge of the influence of the different systems, with their mutual dependancies, would be enlarged; while the individual value of the instruments of our professional operations would be more correctly ascertained.

The following is the only case, that has come to my knowledge, in which so large a dose of the oleum terebinthinæ has been taken, without passing off by stool; and in which so great a quantity passed through the circulation in so short a time.

Having been previously engaged in some experiments on respiration and other physiological subjects, and more lately some articles of the materia medica having occupied my attention, I wished to ascertain by my own feelings the *modus operandi* of a large dose of this medicine. I made a regular register in my common-place book of my feelings at the time of its ingestion, and at the periods mentioned in the following detail, until its effects ceased. As a considerable time may elapse before I can be able to submit to the profession my views on those subjects, in connexion with which the present experiment was made; and as the experience I have had of the benefit resulting from the use of this medicine may lead to its more frequent, but at the same time more cautious, employment; I am induced to lay the following experiment and observations before the readers of the Medical and Physical Journal.

On Tuesday, 7th December, 1820, I swallowed ten drachms of the oleum terebinthinæ in a cup of coffee, at eight o'clock in the morning. I had abstained from supper the preceding night, and immediately before taking it I found myself in robust health; my pulse was 69, soft and equal. At five minutes after its ingestion, pulse 73, rather full, and equal; ten minutes afterwards, 76, and approaching to hard. At the end of half an hour, the pulse is 80, rather harder, and smaller in volume. I feel a slight degree of vertigo and of general chilliness. Occasional eructations of the turpentine vapour, and a sensation at the stomach between that of pain and warmth.

*Nine o'clock*, (at the end of an hour from its ingestion,) the pulse is 82, small, and rather hard; vertigo and chills increased; face very pale, and features shrunk. The pulsation of the carotids is smaller than natural, and gives less resistance. Intellects rather unsteady, and the attention incapable of being long fixed. The vertigo is evidently the effect of diminished flow of blood to the head, and of lessened energy of circulation in the brain. The peculiar sensation at the stomach already alluded to, is now increased and extended in its sphere.

I feel no degree of sickness, but a sensation as if the abdominal viscera were drawn together and towards the spine. In this the abdominal muscles in some degree participate. There is also present some degree of anxiety. Thirst and appetite are considerable; but I have determined to avoid taking any thing as long as possible.

*Half-past nine.*—Pulse 86, and small, but regular; the action of the carotids feeble, and all the animal powers diminished; hand unsteady; my step feeble and rather tottering, especially on walking up stairs, when the weakness is more referrible to the knees. Intellects unsteady in their application to study, and rather confused. Chills greater; but the heat, although lower than natural on the extremities, is higher on the trunk, or at least not below the natural standard in that situation. No perspiration on any part of the body. Appetite remains: no sickness. Considerable thirst, with heat in the stomach, and occasional highly disagreeable eructations.

*Ten o'clock.*—Vertigo and diminished energy of the mental and animal powers continue nearly the same. Pulse 85, and small; anxiety greater; and the feeling of the contents of the abdomen being drawn together and towards the spine, is attended with a sensation quite indescribable, yet it cannot be called pain, although less supportable. It appears to me as if the vital energies were concentrated about the abdominal ganglia and viscera. Appetite considerable, and thirst nearly insupportable.

*Eleven o'clock.*—The foregoing sensations continued to augment; and my determination to leave this medicine to its individual effects could not be longer kept, from the urgency of thirst and appetite: three cups of tea and a slice of toast were therefore taken. No inclination to go to stool. I have determined to wait and observe its effects, without having recourse to any purgative.

These effects continued nearly the same through the greater part of the day; but the vertigo went gradually off after twelve o'clock. At that hour there was present no actual pain, but considerable anxiety, with very slight nausea. Urine nearly the usual quantity, and possessing the violet odour. Still no inclination to go to stool. The feeling of concentrated energy in the abdominal cavity continued; pulse 87, and small. The respired air was loaded with the turpentine odour.

*Four o'clock, afternoon.*—I took only some weak mutton-broth for dinner. Vertigo almost gone. The anxiety and peculiar constricted feeling are rather increased; pulse and other symptoms nearly the same.

*Seven o'clock, evening.*—I drank plentifully of weak tea. The sensation of anxiety and retraction of the abdominal contents nearly the same. Pulse 92, weak and small; occasional eructations. I feel some degree of stupor and confusion of ideas, and consider it a trouble to speak or move. There is great inclination to dose, and, when I indulge it, I soon after awake with a choaking sensation about the glottis, probably induced from the irritation excited in the larynx by the quantity of the turpentine vapour with which the respired air is loaded, and which is now diffused through every apartment in the house, and is even particularly perceptible to myself when I direct the current of respired air through my mouth to my nostrils. Urine much increased in quantity, of the natural colour, and depositing no sediment; the violet odour is present. There is no pain in the region of the kidneys.

*Nine o'clock at night.*—I felt better, and took about two ounces of animal food, and drank some toast-water; but soon afterwards the foregoing disagreeable symptoms and feelings increased considerably.

*Half-past ten o'clock.*—I went to-bed, and afterwards continued to feel still greater anxiety, but no actual pain. A sinking sensation at the præcordia was now present, which continued to increase; and I felt as if falling through the bed and floor. The languor and debility were extreme; and, when I applied my finger to the artery at the wrist, I found my pulse so weak and irregular, and sometimes intermitting, that I was unable to count it. This was also in part owing to a diminished sensation at the extremities of my fingers, as I am at present (the following morning) inclined to suppose. I was considerably alarmed: this produced an irregular palpitation of the heart, and contractions of the muscles of the throat and thorax, somewhat resembling hysteria. I sent for Mrs. C. (who could not sleep in the same room, on account of the strong turpentine odour which filled it.) I requested some sulphuric ether, which was in the room, to be given me. About two tea-spoonsful were taken; and afterwards, so soon as it could be procured, a large draught of water, as hot as it could be swallowed. I felt greatly restored, but dreaded falling asleep. I dosed, however, for five or six hours in a very disturbed manner; and at seven o'clock in the morning found myself much better.

*Eight o'clock in the morning.*—I feel very languid; pulse about 58, and weak. No stool since the ingestion of the oil. Had an enema of warm water only.

*Ten o'clock.*—The enema has remained, and no inclination to stool has been experienced. The turpentine odour is now scarcely sensible. I feel much better, but languid. Pulse 60, weak and soft; hand unsteady; tongue white and dry; urinary organs not affected, but the urine copious, as is to be expected from the quantity of fluids taken. No perspiration. I took my usual breakfast.

In the evening, I felt tolerably well, but languid. My face is, however, of a higher colour at present than natural, which is also in some degree diffused over the body. My bowels are still unmoved.

*Thursday,* (the second day afterwards.)—I am nearly in my usual health; but my bowels have not yet been moved.

*Friday*, (the third day afterwards,) had a very costive motion. In every respect quite well. There is a slight exfoliation of the cuticle on the face, but no where besides.

It had been at one time my intention to have analyzed the products of respiration during the course of this experiment; but the severity of the effects produced from the whole of this fluid having passed through the circulation in so short a space of time as sixteen hours, prevented me from making the inquiry. However, there was abundant proof, quite satisfactory to my mind, that this substance passed from the circulation chiefly by the lungs; although the manner in which I was affected during the period in which these organs were performing that office to the greatest extent, was such as to deprive me of the energy requisite for making this additional inquiry.

Respecting the manner in which this experiment has unexpectedly come in aid of the opinions I have long entertained regarding the more important and more extended office of the lungs in the animal economy than that generally assigned them, I cannot stop here to inquire; nor could room be afforded me at present for such a disquisition. So soon as the facts, experiments, and observations, going to prove the various functions of this organ, and the circumstances by which these functions are modified in health and disease, so as not only to operate changes either in other organs more particularly or upon the whole system; but also to affect, and even entirely to change, the constitution of individuals, and whole races of the human species, according as the modifying causes operate with a confined or extended influence:—so soon as such observations are sufficiently matured, the subject will be more seriously entered upon. In the mean time, I shall content myself with drawing one inference respecting the oleum terebinthinæ, before I pass to the consideration of the practical parts of this article,—viz. that the lungs perform the chief office in ridding the circulation of this substance, when present in a large and noxious quantity.

I have given the detail of this experiment as it was minuted in a common-place book at the time, and it exhibits an account of my feelings at the hours referred to. Both previously to and since it was made, I have employed the oleum terebinthinæ in various diseases; and the practice of a public institution has afforded me more extensive opportunities of proving its efficacy. The following inferences which I shall draw from my experience of its operations upon the animal economy, may not, perhaps, be very different from that which many have believed it to produce in common with other similar substances, when given in a dose of equal strength. I have certainly found it produce effects nearly uniform, when taken internally, if the mode of its exhibition and the circumstances of the individual be taken into consideration. I have tried its effects upon the lower classes of animals; on a few individuals only of those of vermes and insecta; and, so far as the characters of the respective tissues in the highest and in nearly the lowest of the scale of animated nature have a common resemblance, so far the effects assimilate.

1st. I consider that this, like the other essential oils, acts primarily as a stimulant upon the nerves of the texture to which it may be applied, in whatever dose it may be given. This is sufficiently obvious, and has, indeed, been generally allowed.

2dly. When taken internally in a moderate quantity, it excites very slightly the action of the heart immediately after ingestion, by nervous communication between the different ganglia and plexi.

The immediate, but very moderate, action of the heart following its exhibition in a very large dose upon an empty stomach, proves this.

3dly. That the vertigo and intoxicated feeling, (as it is erroneously called,) with diminution of the mental and animal powers, together with the concentrated energy felt in the abdominal viscera, prove that the vital influence of the system and force of the circulation become principally exerted in the viscera in the immediate vicinity of, and in those chiefly supplied with, the ganglial order of nerves, while the other two orders of the nervous system experienced a proportional deficiency; as was shown by the languid circulation and diminished functions of organs depending upon the cerebral and spinal orders of nerves for their supply of nervous influence; and, consequently, that the vertigo, and what has been called the intoxication of the patient, after its ingestion, have arisen from the diminished energy of circulation in the brain. This was proved by my own feelings at the moment; by the shrunk features, pale countenance, and the state of the circulation in the brain, as evinced by the pulsation of the carotids, not in my own case alone, but also in several whom I have narrowly observed while thus affected by this substance.

4thly. It is rapidly absorbed into the circulation, unchanged by the fluids of the digestive canal; and it appears to undergo no change during the circulation of the blood through the various viscera, and is ultimately thrown off by means of the lungs, kidneys, and the secreting and exhalent surfaces; and, during its circulation in the capillary and terminal vessels, it excites their tonicity and action, without inducing any considerable degree of subsequent relaxation.

5thly. When present in the blood in small quantity, it imparts a tonic action to the arterial and capillary vessels, as well as to the heart. But, if it continue to be absorbed into the circulating mass faster than it can be eliminated from it by the different organs destined for that purpose, so that an undue accumulation takes place, as in the instance just detailed, it exhausts or overpowers the irritability of the heart in such a manner as to render this organ incapable of propelling the blood with the requisite energy to the extreme vessels.

6thly. That a large quantity of this substance, when circulating in the mass of fluids, does not always affect the kidneys so severely as has been generally believed.

7thly. In addition to, or perhaps resulting from, its action upon the nervous energy of the part, it acts also upon the insensible contractility or tone of the texture to which it is applied.

(*a.*) Thus it increases the tone and rigidity of muscular fibres.

(*b.*) When applied to the mucous and cellular textures, it gives them the appearance of increased density of structure.

(*c.*) It also diminishes the secretions from the mucous follicles of the digestive tube, by constringing the orifices of the excreting ducts and exhalant vessels; and hence one reason for its uncertainty as a purgative.

(*d.*) It combines with the mucus covering the interior of the intestines, forming with it a gelatinous substance; as I have observed in several instances wherein it has been exhibited as an enema.

(*e.*) If applied to the mucous membrane when deprived of its fine cuticular covering, it gives this tissue a corrugated and more blanched appearance; apparently from constringing its texture and capillary vessels, so as to diminish their calibre and prevent them from admitting the red particles of the blood. It may also, in some degree, combine with this tissue after the manner of tannin, even while it still retains its vitality.

8thly. A large dose of this medicine does not always affect the peristaltic motion of the intestines in such a manner as to cause them to evacuate their contents, unless it be given along with some other purgative.

(*a.*) This may arise from the continued and tonic action it excites in the muscular fibres of the intestines, and which, when induced in the colon, may so act upon the longitudinal bands of fibres collected on the surface of that viscus, in exciting their tonic contraction simultaneously with the like action in its circular fibres, as to throw it into valviform folds, and by these means to obstruct, in the completest manner, the passage of the feces along its canal.

(*b.*) When its purgative effect is produced, it acts entirely by exciting the fibres of the intestinal tube into contraction; while, at the same time, it restrains the secretions from the mucous surface: hence its uncertainty as a purgative when given alone.

(*c.*) Its cathartic effects are more certain, the greater the quantity of feculent matter existing in the prima via at the time of its ingestion. This I have proved, by giving it soon after the bowels have been evacuated by a previous aperient. It has then produced tenesmus, and afterwards even bloody urine, if not followed by an additional purgative. Its more steady action as a cathartic in these circumstances may be explained, by its stimulant effects being chiefly confined to the part with which it comes in contact, which portion, undergoing contraction, pushes the accumulated matters through a successive and more yielding portion of the tube, before a similar degree of contraction can be imparted to it, owing to the intervening, and in some degree consistent, feces preventing the actual contact of this substance in the inferior portion of the canal.

9thly. Its actions on the economy of the higher animals, and its agency as a remedy in disease, are referrible,—1st, to its effects upon the parts to which it is primarily applied, and which, as I have already noticed, may vary according to the constitution and texture of the part; 2d, to its effects by means of nervous communication and continuity of tissue, which are very confined; 3d, according to its presence in the circulating fluids, and the quantity of it existing in the blood within a given period; and 4th, to its effects as they may result from the simultaneous actions of the three preceding,—namely, the effects arising from its local influence and resulting sympathies, and from its presence in the circulating mass of fluid.

10thly. This substance appears to act in a nearly similar manner upon animals of the classes insecta and vermes, (from the few trials I have made;) and according to the proportion existing between the nervous system of the animal and the dose. But the effect is greatly modified by the texture to which it is applied, and according as it may come in immediate contact with their respiratory organs.

(*a.*) When applied to these organs in sufficient quantity, it acts by producing a mechanical impediment to changes induced by the air.

(*b.*) It also constringes the texture and orifices of their respiratory organs.

(*c.*) In addition to its effects upon the nervous system and respiratory organs of those animals, it produces an astringent effect upon their soft and gelatinous tissues, apparently from combining in part with its texture, and preventing the evolution of its functions.

Having shown its effects upon the animal economy, according as my opportunities have afforded me the imperfect knowledge,

I would have pursued the subject, by making some observations respecting its employment as a remedy of no mean efficacy in various diseases; but, as I might be led to a tedious length, I shall defer the fulfilment of this intention until the following Number of the Journal.

*Walworth Terrace; May* 1821.

